# Genomic and exoproteomic analyses of cold‐ and alkaline‐adapted bacteria reveal an abundance of secreted subtilisin‐like proteases

**DOI:** 10.1111/1751-7915.12343

**Published:** 2016-02-01

**Authors:** Jeanette E. Lylloff, Lea B.S. Hansen, Morten Jepsen, Kristian W. Sanggaard, Jan K. Vester, Jan J. Enghild, Søren J. Sørensen, Peter Stougaard, Mikkel A. Glaring

**Affiliations:** ^1^ Department of Plant and Environmental SciencesUniversity of CopenhagenThorvaldsensvej 40Frederiksberg1871Denmark; ^2^ Department of BiologyUniversity of CopenhagenCopenhagenDenmark; ^3^ Interdisciplinary Nanoscience Center and Department of Molecular Biology and GeneticsAarhus UniversityAarhusDenmark

## Abstract

Proteases active at low temperature or high pH are used in many commercial applications, including the detergent, food and feed industries, and bacteria specifically adapted to these conditions are a potential source of novel proteases. Environments combining these two extremes are very rare, but offer the promise of proteases ideally suited to work at both high pH and low temperature. In this report, bacteria from two cold and alkaline environments, the ikaite columns in Greenland and alkaline ponds in the McMurdo Dry Valley region, Antarctica, were screened for extracellular protease activity. Two isolates, *Arsukibacterium ikkense* from Greenland and a related strain, *Arsukibacterium* sp. MJ3, from Antarctica, were further characterized with respect to protease production. Genome sequencing identified a range of potential extracellular proteases including a number of putative secreted subtilisins. An extensive liquid chromatography–tandem mass spectrometry analysis of proteins secreted by *A. ikkense* identified six subtilisin‐like proteases as abundant components of the exoproteome in addition to other peptidases potentially involved in complete degradation of extracellular protein. Screening of *Arsukibacterium* genome libraries in *Escherichia coli* identified two orthologous secreted subtilisins active at pH 10 and 20°C, which were also present in the *A. ikkense* exoproteome. Recombinant production of both proteases confirmed the observed activity.

## Introduction

Proteases are enzymes capable of hydrolysing peptide bonds and are used as such in a variety of commercial applications, including the food and feed, textile, leather, paper and detergent industries. They represent one of the single most important groups of industrial enzymes in terms of worldwide sales with the detergent proteases being the most prominent (Gupta *et al*., 2002; Maurer, 2004). The currently used detergent proteases are from the subtilisin family of serine proteases. Members of this family have been identified in many organisms, but despite their widespread occurrence, the detergent subtilisins have all been isolated from *Bacillus* species. These enzymes are mainly of the high‐alkaline type of subtilisins, extracellular proteases that are active and stable at pH values up to 12, making them suitable for detergent applications (Fujinami and Fujisawa, 2010). In addition, both thermostability and activity at low temperature are desirable properties for detergent proteases in, e.g. dishwashing and cold water‐laundry processes. Thermostable enzymes for application in high‐temperature processes have been developed from thermophilic *Bacillus*, whereas cold‐adapted subtilisins have been successfully engineered based on known *Bacillus* subtilisins (Wintrode *et al*., 2000; Tindbaek *et al*., 2004; Siddiqui *et al*., 2009).

As an alternative to protein engineering of existing enzymes, identification of novel proteases from cold‐ or alkaline‐adapted organisms has been the focus of many studies (Kasana, 2010; Niehaus *et al*., 2011; Kuddus and Ramteke, 2012). Natural alkaline environments, such as soda lakes and alkaline soils, harbour microbial life that produces extracellular enzymes adapted to these conditions. Similarly, enzymes adapted to low temperature may be isolated from organisms living in permanently cold environments such as the polar and high alpine regions and the deep sea (Fujinami and Fujisawa, 2010; Margesin and Feller, 2010; Cavicchioli *et al*., 2012). Bioprospecting in these extreme environments thus offers new opportunities for isolation of novel proteases active at high pH and/or low temperature. Such proteases might be immediately useful in specific applications or they may yield insight into the mechanisms of activity and form the basis for protein engineering studies aimed at improving the performance of existing enzymes.

Permanently cold and alkaline habitats are very rare on Earth and only a few such environments have been described. One is the submarine ikaite columns in the Ikka Fjord in Southern Greenland. The columns are formed by precipitation of calcium carbonate from the mixture of seawater and alkaline spring water, and the interior of the columns is permanently cold (less than 6°C) and alkaline (above pH 10) (Buchardt *et al*., 1997; Hansen *et al*., 2011). It has previously been demonstrated that the columns are home to a diverse bacterial community adapted to these conditions (Schmidt *et al*., 2006; Vester *et al*., 2013) and a potential source of novel enzymes (Schmidt and Stougaard, 2010; Vester *et al*., 2014, 2015; Glaring *et al*., 2015). Similar extreme conditions can be found in a series of alkaline ponds with pH values above 10 located in the McMurdo Dry Valley region, Antarctica (Jungblut *et al*., 2012).

The biotechnological potential of secreted proteases produced by bacteria adapted to both cold and alkaline conditions, prompted us to perform a screen for protease‐producing isolates from both the ikaite columns in Greenland and the Antarctic alkaline ponds. Two related strains, one from each location, were isolated and characterized with respect to protease production. Genome sequencing coupled with sensitive proteome analyses identified a large number of proteases secreted to the growth medium, including a range of subtilisin homologues. Expression and characterization of two orthologous extracellular subtilisin‐like proteases in *Escherichia coli* verified the observed cold‐ and alkaline‐active properties, suggesting that these environments could be a source of future industrial proteases for low temperature and/or alkaline processes.

## Results and discussion

### Screening for isolates producing extracellular protease activity

Protease‐producing bacterial isolates were identified by screening on solid media containing the chromogenic substrate azurine‐cross‐linked (AZCL) casein at pH 10 and a temperature of 5–15°C. Screening of ikaite column isolates was performed on a previously established collection of strains (Schmidt *et al*., 2006) and resulted in several closely related isolates producing extracellular proteases active at pH 10 and temperatures as low as 5°C (data not shown). One of these isolates was previously described as the Gammaproteobacterium *Arsukibacterium ikkense* (Schmidt *et al*., 2007) and it has been demonstrated that proteolytic activities present in the culture medium are able to produce bioactive peptides from casein at temperatures as low as 5°C (De Gobba *et al*., 2014). The type strain (GCM72) was used for further studies of the observed protease activity.

Samples from the Antarctic alkaline ponds were screened by direct plating of sample material onto screening plates. One isolate producing high levels of extracellular protease active at pH 10 and 10°C was picked out and further characterized. Interestingly, the sequence of the 16S rRNA gene was 98.6% identical over 1440 bp to *A*. *ikkense* isolated from the ikaite columns, suggesting that these strains, isolated from similar environments at the two poles, are closely related and might represent the same genus or species. The Antarctic isolate was named *Arsukibacterium* sp. MJ3 (termed MJ3) to represent this observation. Both *A. ikkense* and MJ3 were also closely related to *Rheinheimera perlucida* (99.4% and 98.2% identity respectively), a marine bacterium isolated from the surface waters of the Baltic Sea (Brettar *et al*., 2006). Further characterization will be needed to determine the phylogenetic relationship between the alkaline‐adapted *Arsukibacterium* and the *Rheinheimera* genus.

### Characterization of protease‐producing isolates

The pH and temperature range for growth of *A. ikkense* was previously determined to be pH 7.5–10 with optimum at pH 9.2–10 and 0–30°C with optimum at 15°C respectively (Schmidt *et al*., 2007). A similar analysis of isolate MJ3 showed growth at 5–15°C in liquid media with optimal growth at 15°C and little or no growth at 20°C, making this a truly psychrophilic isolate. Optimal growth was observed at pH 9–10, with no growth at pH 8 (Fig. S1). The Antarctic isolate MJ3 is thus more restricted in its growth range than *A. ikkense* and more specifically adapted to cold and alkaline conditions.

**Figure 1:**
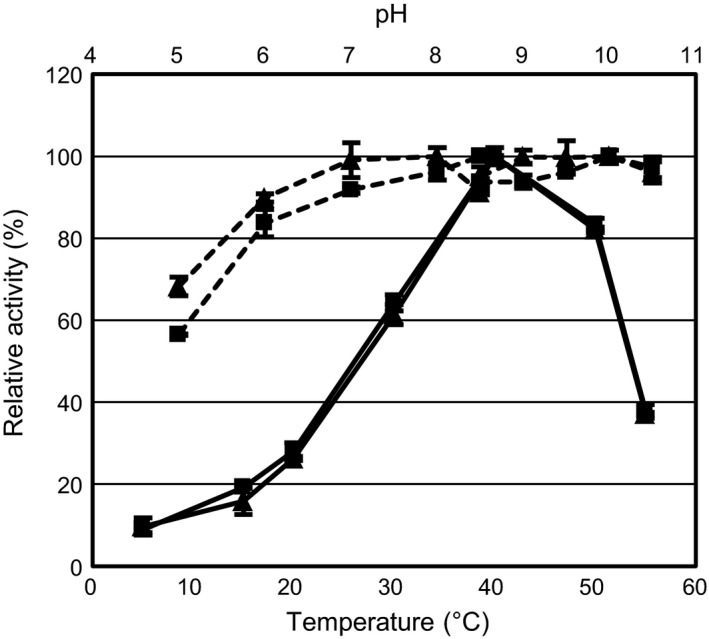
Temperature and pH optimum of total extracellular protease activity in growth medium from *Arsukibacterium ikkense* (squares) and *Arsukibacterium* sp. MJ3 (triangles). Temperature assays (solid lines) were carried out in glycine buffer pH 9.5. The pH assays (dashed lines) were incubated at 30°C in a three‐component buffer (pH 5–8.5) or a glycine buffer (pH 8.5–10.5). The maximum activity observed in the temperature assay and each of the two buffer assays was set to 100%. Values are given as the average of three measurements (±SD).

Extracellular protease activity was produced on solid media at all growth temperatures for both isolates. An analysis of the total protease activity secreted to the growth medium in liquid cultures grown at 15°C and pH 10 revealed a temperature range of activity of 5–55°C with an optimum around 40°C and approximately 25% activity retained at 20°C for both isolates. The pH range of activity was wide, with high relative activity from pH 6–10.5 for both isolates (Fig. 1). The similar activity profiles indicate a significant overlap in the complement of extracellular proteases produced by the two related isolates, although it is worth noting that some proteases may not be active on the casein substrate. Previous studies of bacterial isolates producing cold‐ and/or alkaline‐active protease have mainly focussed on single activities, frequently by direct purification of the enzyme from culture medium (Kulakova *et al*., 1999; Zeng *et al*., 2003; Wang *et al*., 2005; Zhu *et al*., 2009). Genome sequences of various *Bacillus* species have been found to encode a range of proteases of the subtilisin type and the alkaliphilic *Bacillus* sp. strain KSM‐LD1 contains a record number of 11 putative extracellular subtilisins (Takimura *et al*., 2007). It is thus likely that the observed extracellular protease activity is the result of multiple secreted endoproteases.

### Genome sequencing and identification of putative secreted proteases

In order to identify putative secreted proteases, draft genome sequences were prepared from both isolates. This resulted in a 4.14 Mb genome in 89 contigs for *A. ikkense* and a 3.75 Mb genome in 196 contigs for isolate MJ3 (Table S1). Following gene‐calling, the predicted coding sequences (CDSs) were annotated by comparison to the Pfam database (http://pfam.sanger.ac.uk) and all CDSs assigned to protease‐containing protein families as defined by the MEROPS peptidase database (Rawlings *et al*., 2012) were further analysed. This resulted in 188 and 157 CDSs from *A. ikkense* and MJ3, respectively, with significant hits (E‐value < 1e‐05) to protease‐containing Pfam families (Table S2). As these families may contain other catalytic activities some of the hits are likely to encode non‐proteolytic enzymes. The difference in the number of predicted proteases could be a consequence of the smaller draft genome size of MJ3. Compared to the total number of predicted CDSs (Table S1), the relative frequency of proteases in the two genomes was similar with 4.9% and 4.6% found for *A. ikkense* and *MJ3* respectively.

The putative subcellular localization of the identified proteases was predicted by PSORTb for Gram‐negative bacteria (Table S2). For *A. ikkense*, 12 proteases were predicted to be extracellular of which six were from peptidase family S8, which is based on the serine endopeptidase subtilisin, all with a conserved catalytic triad of aspartic acid, histidine and serine. Four of these also carried a potential signal peptide as predicted by SignalP, making it likely that these are indeed extracellular proteases. Five proteases were predicted to be outer membrane associated, but their predicted MEROPS families (M14, M16, M23 and S41) are primarily involved in peptidoglycan degradation and protein processing. PSORTb was unable to predict the localization for 73 proteases of which 35 contained a potential signal peptide. These could potentially all represent secreted proteases and among them were three additional subtilisin homologues, a trypsin‐like protease (MEROPS family S1), and a family M9 collagenase. Isolate MJ3 contained five PSORTb predicted extracellular proteases. Four of these were subtilisin homologues with a conserved catalytic triad and three of them included a SignalP predicted signal peptide. Among 61 proteases with unknown localization were 36 with a predicted signal peptide including two subtilisin homologues and a trypsin‐like protease (Table S2). Approximately, one‐third of all the protease CDSs carried a predicted signal peptide (Table S2), which is in agreement with previous studies of proteolytic enzymes in bacterial genomes (Methé *et al*., 2005).

### Mass spectrometry based identification of secreted proteases

The total complement of extracellular proteins is referred to as the exoproteome and contains both actively secreted proteins, as well as proteins that are released to the growth medium due to cell lysis or during normal cellular metabolism (Armengaud *et al*., 2012). Given the close relationship between the two *Arsukibacterium* isolates and that the *A. ikkense* draft genome encoded the highest number of identifiable putative secreted proteases (Table S2), this isolate was chosen for exoproteome analysis. The extracellular proteases potentially responsible for the observed proteolytic activity were identified by liquid chromatography–tandem mass spectrometry (LC‐MS/MS) analyses of growth medium from actively secreting cultures of *A. ikkense*. Samples of the growth medium from cultures growing at 15°C and 5°C were taken at the end of the exponential phase of growth (sample A and C respectively) and additional samples of cultures growing at 5°C were taken in the middle of the exponential phase of growth (sample B) and 72 h into the stationary phase (sample D).

The precipitated proteins were denatured and digested with trypsin prior to LC‐MS/MS analyses of three technical replicates of each of the four samples. The resulting data were used to interrogate a database containing CDSs predicted from the *A. ikkense* genome. A total of 901 proteins, corresponding to 23.6% of all CDSs, were detected in the growth medium from all samples (data not shown). In addition to confirming the sensitivity of the method, the high number of detected proteins suggests that intracellular proteins are present in the medium, e.g. due to cell death and lysis. Studies of *Bacillus subtilis* have shown that normal cellular metabolism, such as protein and cell wall turnover, and even non‐classical secretion mechanisms, also contribute to shaping the exoproteome (Tjalsma *et al*., 2004; Yang *et al*., 2011a). To minimize the number of low‐abundance proteins and potential false‐positive detections, only protein identifications based on at least two unique peptide observations in each of the three technical replicates were accepted (Table S3). This resulted in a list of 458 proteins. The abundance of each protein was estimated by a semi‐quantitative approach based on spectral counting. A detailed list of identified proteins in all replicates is given in Table S3, including Mascot score, spectral count and normalized abundances.

Among the 458 detected extracellular proteins were 60 putative proteases (Table S4). The analysis thus identified almost one‐third of the 188 predicted protease‐encoding sequences, underlining the depth of the proteome analysis. The highest total abundance was observed in the samples taken at the end of the exponential phase of growth making up 11.7% and 15% of the exoproteome at 5 and 15°C respectively (Table 1, samples A and C). Given the sensitivity of the MS method, this may include many cell‐associated or intracellular proteases released to the culture medium due to cell lysis. In addition to the proteases, the exoproteome was dominated by flagellar and pilus proteins, transporters/receptors and uptake systems, as well as stress response and chaperone functions (Table S3). The most abundant protein was a CspA family cold shock protein, which made up almost 5% of the exoproteome at 5°C. Identification of several abundant ribosomal proteins confirmed that cell lysis is an important contributor of proteins to the exoproteome. It is worth noting that many of the apparently cytoplasmic proteins abundant in the exoproteome (e.g. superoxide dismutase, malate dehydrogenase and elongation factors) have also been identified by exoproteome analysis of *Bacillus subtilis* (Tjalsma *et al*., 2004).

**Table 1:** The most abundant proteases detected in the *A. ikkense* growth medium by LC‐MS/MS (≥ 0.25% in any sample)

Sequence	Predicted function	Mass (kDa)	MEROPS id	PSORT	SignalP	A (15°C)		B (5°C)		C (5°C)		D (5°C)	
						Abd.	STD	Abd.	STD	Abd.	STD	Abd.	STD
peg.2527	Alkaline serine protease	79.075	S8	Extracellular	N	0.8%	0.0	0.2%	0.0	2.4%	0.3	0.9%	0.1
peg.1689	Alkaline serine protease	79.208	S8	Extracellular	Y	0.6%	0.0	0.2%	0.0	1.8%	0.3	0.8%	0.1
peg.2189	Dipeptidyl carboxypeptidase Dcp	80.473	M3	Unknown	Y	1.2%	0.1	0.2%	0.0	0.8%	0.2	0.6%	0.1
peg.2262	Cold‐active alkaline serine protease	56.646	S8	Unknown	Y	0.5%	0.0	0.1%	0.0	1.5%	0.1	0.7%	0.1
peg.2438	Cyanophycinase	49.102	S51	Extracellular	Y	0.2%	0.0			1.0%	0.2	0.4%	0.1
peg.1776	Alkaline serine protease	53.843	S8, I9	Extracellular	Y	0.5%	0.0			0.8%	0.0	0.4%	0.1
peg.252	Prolyl endopeptidase	79.351	S9	Periplasmic	Y	0.9%	0.1	0.1%	0.0	0.3%	0.1	0.3%	0.0
peg.510	Serine protease, subtilase family	86.855	S8	Extracellular	Y	0.3%	0.0			0.9%	0.1	0.2%	0.0
peg.3495	Peptidase B	46.278	M17	Cytoplasmic	N	0.8%	0.1					0.1%	0.0
peg.2018	Aminopeptidase	47.965	M28, M20	Extracellular	Y	0.4%	0.0			0.4%	0.0	0.1%	0.0
peg.3545	Carboxyl‐terminal protease	47.540	S41	Outer membrane	N	0.1%	0.0			0.4%	0.0	0.3%	0.1
peg.99	Microbial collagenase, secreted	104.40	M9	Extracellular	Y			0.0%	0.0	0.6%	0.0	0.1%	0.0
peg.107	Proline dipeptidase	43.84	M24	Cytoplasmic	N	0.7%	0.0						
peg.2612	Peptidase, M13 family	76.412	M13	Unknown	Y	0.4%	0.1	0.1%	0.0			0.1%	0.0
peg.1843	Protease II	80.565	S9	Periplasmic	Y	0.5%	0.0	0.1%	0.0			0.1%	0.0
peg.1001	Hypothetical protein	59.03	S41	Extracellular	N	0.2%	0.0			0.3%	0.1	0.2%	0.1
peg.893	Xaa‐Pro dipeptidase PepQ	50.338	M24	Cytoplasmic	N	0.6%	0.0						
peg.1350	Aminoacyl‐histidine dipeptidase	52.645	M20	Cytoplasmic	N	0.5%	0.1					0.1%	0.0
peg.1064	Microsomal dipeptidase	43.675	M19	Unknown	Y	0.6%	0.1	0.0%	0.0				
peg.3769	Serine protease, subtilase family	128.14	S8	Unknown	Y	0.2%	0.0	0.0%	0.0	0.3%	0.1	0.1%	0.0
peg.2515	Oligopeptidase A	77.377	M3	Cytoplasmic	N	0.5%	0.0						
peg.2856	Alanyl dipeptidyl peptidase	75.712	S9, S15	Unknown	Y	0.4%	0.0	0.0%	0.0				
peg.1846	Protease III precursor	107.90	M16	Unknown	N	0.4%	0.0	0.0%	0.0				
peg.867	Phospholipid‐binding protein	17.424	I51	Periplasmic	N	0.4%	0.0						
peg.3630	Peptidase	61.109	M28	Unknown	N	0.3%	0.0					0.1%	0.0
peg.2900	Dipeptidyl peptidase IV	93.681	S9, S15	Unknown	Y	0.3%	0.0					0.0%	0.0
peg.96	Dipeptidyl peptidase IV	85.803	S9	Unknown	Y	0.3%	0.0						
Other proteases						2.4%		1.2%		0.1%		0.5%	
Total number of proteases detected						46		30		14		28	
Total abundance						15.0%		2.2%		11.7%		6.1%	

Abundance (Abd.) is given as the average of three technical replicates for each sample (±SD). The functional predictions were taken from the RAST server annotations and the protease family was defined by the Pfam domain matches and listed using the MEROPS peptidase database classification (MEROPS id). PSORTb localization and signal peptide prediction by SignalP (Yes/No) was performed as described in the text. Samples of cultures growing at 5 and 15°C were taken as follows: Samples A and C, end of the exponential phase of growth; Sample B, middle of the exponential phase; Sample D, 72 h into the stationary phase.

Among the 60 proteases identified in the exoproteome, PSORTb failed to predict the localization of 24 proteases and predicted another 20 to be cytoplasmic or cytoplasmic membrane‐localized. Almost half of the putative secreted proteases did not contain a discernible signal peptide (Table S4), clearly highlighting the problems of accurately predicting the cellular localization of proteins. The proteases responsible for the observed extracellular endoproteolytic activity are likely to be relatively abundant compared to leaked intracellular proteins and among the most abundant extracellular proteases (≥ 0.25% in any sample) were six subtilisin homologues of varying size (Table 1). A number of other abundant proteases were categorized as oligo‐, di‐ or exopeptidases and some of these may play a nutritional role by acting in concert with the endoproteolytic subtilisins to ensure complete degradation of extracellular proteins. In addition to the subtilisin homologues, a relatively abundant protease annotated as a family M9 secreted collagenase was only detected in cultures grown at 5°C. If the natural substrate of this protease is a hydroxyproline‐rich protein, such as those found in collagen and in the cell walls of algae and plants, then the dye‐linked casein used for measuring protease activity may not be a suitable substrate for this enzyme. Similarly, the identified exopeptidases are not expected to be active on this substrate and will thus not contribute to the total protease activity measured in the growth medium.

### Sequence analysis of the subtilisin‐like proteases

**Figure 2:**
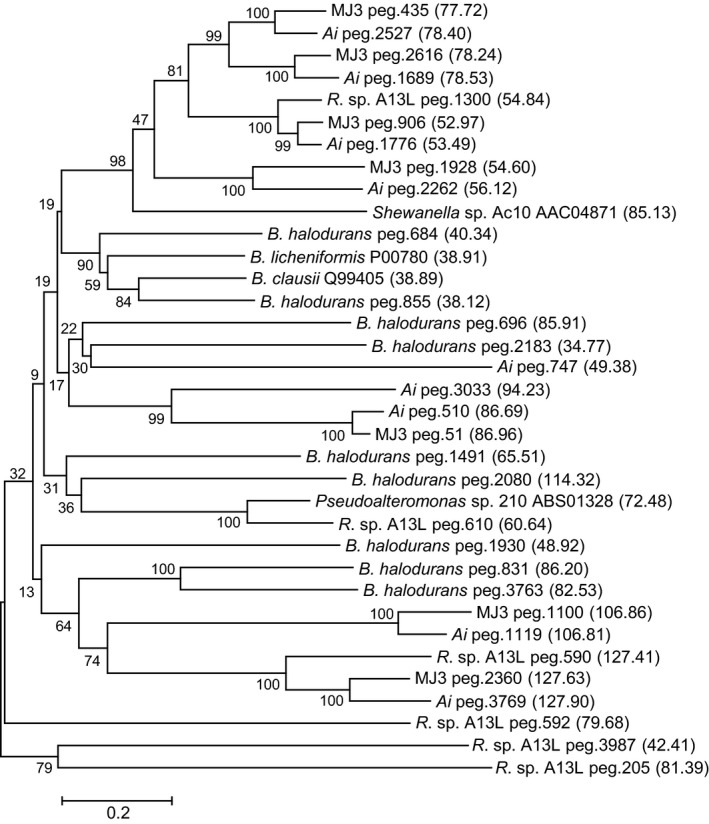
Neighbour‐joining phylogenetic tree of the core catalytic domain of predicted subtilisin‐like proteases from the genomes of *Arsukibacterium ikkense* (Ai) and *Arsukibacterium* sp. MJ3. Included are 9 sequences from the genome of the alkaliphilic *Bacillus halodurans* strain C125, 6 sequences from the genome of the related, alkaline‐adapted *Rheinheimera* sp. A13L, two sequences of characterized, cold‐adapted subtilisins (Kulakova *et al*., 1999; Acevedo *et al*., 2008), the type protease of family S8A, subtilisin Carlsberg from *Bacillus licheniformis*, and the high‐alkaline subtilisin M‐protease from *Bacillus clausii*. The underlying alignment was trimmed corresponding to residue 132‐377 in subtilisin Carlsberg. Sequence IDs are given as the RAST server numbering (peg) or GenBank accession number. The molecular weight of the complete ORF is given in kDa in parenthesis.

The subtilisin‐like proteases from peptidase family S8 were identified as a major component of the exoproteome of *A. ikkense* by MS analysis (Table 1). Microbial extracellular subtilisins are non‐specific serine endopeptidases (Siezen and Leunissen, 1997) and one or more of the identified subtilisin homologues are likely to be responsible for the protease activity observed at low temperature and high pH. A total of nine and seven putative family S8 proteases were encoded in the genomes of *A. ikkense* and MJ3, respectively, in good agreement with a previous analysis of *Bacillus* genomes (Takimura *et al*., 2007). Comparisons between the predicted subtilisins from the two isolates revealed seven closely related pairs (65–86% identity, Fig. 2) and the seven subtilisin homologues identified in the *A. ikkense* exoproteome (Table S4) were all from these orthologous pairs.

Genome mining of available genome sequences from the related *Rheinheimera* sp. A13L isolated from an alkaline lake (Gupta *et al*., 2011) and the alkaliphilic *Bacillus halodurans* strain C125 (Takami *et al*., 2000) identified six and nine subtilisin‐like proteases respectively (data not shown). Alignment of the core catalytic domains with sequences from *A. ikkense* and MJ3 did not reveal any clear groups of homologous domains representing all species (Fig. 2). In related isolate *R*. sp. A13L, only two sequences clustered with *A. ikkense* and MJ3 and the remaining four had no obvious close homologues in the polar isolates. The finding that secreted proteases from the cold‐ and alkaline‐adapted isolates appear to be distinct from those found in a related alkaline‐adapted isolate increases the possibility of finding novel activities among these proteases.

### Cloning and characterization of two orthologous subtilisin‐like proteases

In order to identify individual proteases that could be actively expressed and secreted by *E. coli* without extensive optimization, genome libraries of both strains were constructed and clones showing extracellular protease activity at 20°C and pH 10 were isolated. Sequencing revealed that the active clones from both libraries covered the same genomic region containing a single putative secreted serine protease of the subtilisin type (CDS peg.1776 and peg.906 from *A. ikkense* and MJ3 respectively). The genomic regions from the two isolates were highly syntenic and the two proteases were 86% identical (91% similarity) suggesting that they are orthologues serving a similar function in the two strains. The *A. ikkense* orthologue (peg.1776) was present as an abundant protein at the end of the exponential phase of growth at both 5 and 15°C (Table 1) and both proteases contained an N‐terminal inhibitor domain from MEROPS family I9 and a C‐terminal pre‐peptidase domain (Pfam PF04151), indicating significant processing of these protease during secretion.

**Figure 3:**
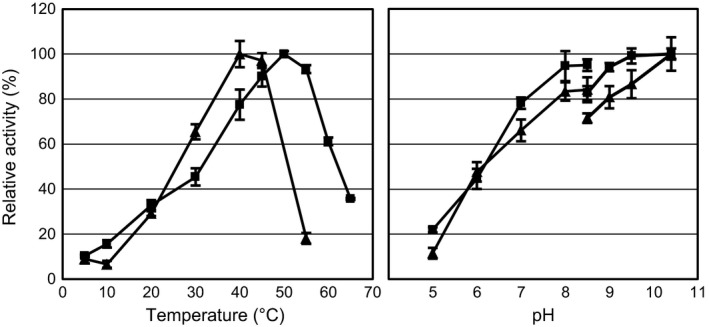
Temperature and pH optimum of the recombinant subtilisin‐like proteases peg.1776 from *Arsukibacterium ikkense* (squares) and peg.906 from *Arsukibacterium* sp. MJ3 (triangles). Temperature assays (left) were carried out at pH 9.5 and pH assays (right) were incubated at 40–45°C in a three‐component buffer (pH 5–8.5) or a glycine buffer (pH 8.5–10.5). The maximum activity observed in the two assays was set to 100%. Values are given as the average of three measurements (±SD).

The two orthologous subtilisin‐like proteases were cloned with a C‐terminal His‐tag and expressed in *E. coli*. Following induction, protease activity was detected in the growth medium, which was subsequently concentrated by ultrafiltration and subjected to affinity purification. Protease yield in *E. coli* was generally in the microgram range per litre of culture medium, with the highest yield obtained for the *A. ikkense* protease. Industrial production of bacterial alkaline proteases is normally carried out in optimized *Bacillus* fermentation systems and optimal production of the *Arsukibacterium* proteases would likely require substantial optimization and redesign of the expression system, perhaps utilizing a different expression host. The purified proteases were characterized with respect to temperature and pH optimum (Fig. 3). The pH profiles for the two proteases were similar and maximum activity was observed in assays at pH 10.4, which was the highest pH tested. It is possible that the proteases are active at higher pH values, similar to the high‐alkaline subtilisins (Saeki *et al*., 2007). The high relative activity at neutral pH measured in the growth medium (Fig. 1) would also suggest that both isolates produce additional extracellular proteases with optimal activity at lower pH. Despite the high similarity, the optimal temperature differed between the two proteases with maximum activity at 50°C and 40°C for *A. ikkense* peg.1776 and MJ3 peg.906, respectively, suggesting a distinct adaptation to lower temperature in the MJ3 protease. The temperature optimum of the *A. ikkense* protease was higher than the optimum determined for the growth medium (Fig. 1) suggesting that other proteases are responsible for a significant part of the extracellular protease activity produced by this strain. Both proteases retained approximately 30% activity at 20°C.

Many cold‐active proteases have previously been isolated from cold‐adapted bacteria living at near‐neutral pH (Joshi and Satyanarayana, 2013). This includes a few subtilisin‐type proteases with temperature optima similar to those reported here, but most of these subtilisins display pH optima lower than the characterized *Arsukibacterium* subtilisins (Wang *et al*., 2005, 2008; Zhu *et al*., 2009; Yang *et al*., 2011b). High‐alkaline subtilisins have been isolated from alkaliphilic bacteria, primarily species of *Bacillus*, and although special exceptions do exist, they generally have temperature optima above 60°C (Okuda *et al*., 2004; Kobayashi *et al*., 2007). The two recombinant proteases from *Arsukibacterium* may not be novel in terms of temperature optimum alone, but the high‐alkaline activity coupled with temperature optima that are lower than previously reported for this type of protease, could indicate the presence of adaptations as a consequence of the cold and alkaline environment from which they were isolated. Additional work will be required to determine whether enzymes with similar properties can be found among the other subtilisin‐like proteases identified in the genome and exoproteome analyses. In addition to the subtilisins, many of the other identified proteases could potentially be adapted to lower temperatures and high pH. Due to the unique polyextreme environmental conditions in which they are active, this may include low‐temperature enzymes for which there are no known alkaline‐active forms and vice versa. Continued identification and characterization of these proteases will increase our understanding of the molecular adaptations that allow the enzymes to be active under these conditions.

## Conclusion

Two related isolates of the *Arsukibacterium* genus were identified as major extracellular protease producers from two cold and alkaline environments. Genome sequencing coupled with extensive exoproteome analysis identified a range of secreted subtilisin‐like proteases and the observed extracellular activity is likely to be a result of several endoproteolytic activities. Cloning and purification of an orthologous pair of proteases confirmed the cold‐ and alkaline‐active properties. The results establish isolates of the *Arsukibacterium* genus as prominent protease producers capable of secreting a diverse array of subtilisin homologues and other oligo‐, di‐ and exopeptidases. Importantly, this study supports the notion that the permanently cold and alkaline environments could be a source of future industrial proteases. Such enzymes may be unique in their molecular adaptations to the rare environmental combination of low temperature and high pH found in the ikaite columns and the Antarctic alkaline ponds and may consequently form the basis for protein engineering studies aimed at improving the performance of existing proteases for, e.g. the detergent industry.

## Experimental procedures

### Growth, screening and characterization of protease‐producing bacterial isolates

All isolates were grown on standard R2 medium with or without 15 g/l agar for solid plates (R2A) and liquid medium (R2B) respectively. R2 medium was adjusted to pH 10 by either 20 mM (R2A) or 100 mM (R2B) carbonate/bicarbonate buffer. An additional 1% NaCl was added to all media and R2B was further supplemented with a solution of trace elements and vitamins as described (Schmidt *et al*., 2007). Screening for extracellular protease activity was performed on R2A pH 10 with 0.5 mg/ml AZCL casein (Megazyme, Bray, Ireland) and no glucose. Screening was performed on either pure isolates (for the ikaite columns) or by direct plating of environmental samples (for the Antarctic ponds). Plates were incubated at 5–15°C and activity was scored over a period of several weeks. Protease‐producing isolates were re‐streaked at least twice to ensure purity and to confirm the protease‐producing phenotype. Protease activity in culture supernatants was determined as described below. Isolates were characterized phylogenetically by amplification of 16S rDNA using the standard primers 27F and 1492R and subsequent sequencing of the resulting PCR product using the same primers. The 16S rRNA gene sequence from *Arsukibacterium* sp. MJ3 has been deposited in GenBank under accession number KT388746. The optimal conditions for growth of *Arsukibacterium* sp. MJ3 was investigated on R2A and R2B at pH 10 and 15°C for optimal temperature and pH respectively.

### Genome sequencing and analysis

Genomic DNA was extracted using the Gentra Puregene Yeast/Bact. Kit (QIAGEN Nordic, Copenhagen, Denmark) according to the manufacturer's instructions for Gram‐negative bacteria. Genome data were obtained by 2 × 100 bp paired‐end sequencing of 500 bp insert libraries on an Illumina HiSeq system. After trimming and quality‐filtering, contigs were assembled using IDBA‐UD (Peng *et al*., 2012) and CLC Genomics Workbench (CLCbio, Aarhus, Denmark). Gene‐calling and annotation of the resulting contigs were performed using the Rapid Annotation using Subsystem Technology server (http://rast.nmpdr.org/rast.cgi) (Aziz *et al*., 2008). Updated versions of the assembled genome sequences are available from GenBank under accession numbers LAHO00000000 for *A. ikkense* GCM72 and LAHP00000000 for *Arsukibacterium* sp. MJ3 (Lylloff *et al*., 2015).

Predicted CDSs were functionally annotated by a HMMER search (http://hmmer.org/) against a Pfam protein domain database (http://pfam.sanger.ac.uk) and CDSs assigned to one of the protease‐containing protein families as defined by the MEROPS peptidase database cross‐links (http://merops.sanger.ac.uk/) were isolated (Rawlings *et al*., 2012). All hits with an E‐value < 1.0e‐5 were discarded before further analysis. The nucleotide sequences of all predicted protease ORFs are available in Table S2. Potential signal peptides were identified using SignalP 4.1 (http://www.cbs.dtu.dk/services/SignalP/) for Gram‐negative bacteria with default options (Petersen *et al*., 2011). Additionally, protein localization was predicted by PSORTb version 3.0.2 (http://www.psort.org/) (Yu *et al*., 2010). Sequences were handled and analysed using Biopieces (http://www.biopieces.org/) and CLC Main Workbench (CLCbio). Alignments and phylogenetic trees were constructed and visualized using MEGA (http://www.megasoftware.net/).

### Exoproteome analysis by LC‐MS/MS

Three independent cultures of *A. ikkense* were grown in R2B without glucose at 15°C and 5°C. Samples for exoproteome analysis by MS were taken at 15°C at the end of the exponential phase of growth [approximate optical density (OD) at 600 nm of 2.0, sample A] and at 5°C in the exponential phase of growth (OD of 1.0, sample B), at the end of the exponential phase (approximate OD of 2.0, sample C) and 72 h into the stationary phase (sample D). Duplicate samples of 2 ml growth medium from each of the three cultures were harvested by two rounds of centrifugation to remove cells and cell debris. The six samples from each of the four time points were pooled, precipitated in 20% TCA and subsequently divided into three technical replicates. The precipitated proteins were denatured, reduced by treatment with 5 mM DTT, alkylated with 25 mM iodoacetamide (IAA) and finally digested with trypsin. LC‐MS/MS analyses were performed on an Easy‐nLC II nanoHPLC (Thermo Scientific, Hvidovre, Denmark) connected in‐line to a TripleTOF 5600+ mass spectrometer (AB Sciex, Framingham, MA, USA) equipped with a NanoSpray III source (AB Sciex). Peptides were eluted from the analytic column (15 cm length, 75 μm internal diameter, and packed with ReproSil‐Pur C18‐AQ 3 μm resin) using a 50 min gradient from 5% to 35% solvent B (0.1% formic acid, 90% acetonitrile) at a flow rate of 250 nl/min.

The collected MS data were converted to Mascot generic format and the resulting files were used to interrogate the database containing predicted CDSs from the *A. Ikkense* genome. The analyses were performed using the Mascot search engine (Matrix Science, Boston, MA, USA) with the following settings: Up to one missed trypsin cleavage allowed, carbamidomethyl as fixed modification, methionine oxidation as variable modification, a peptide mass tolerance offset of 15 ppm and a fragment mass tolerance of 0.2 Dalton. For acceptance of peptide matches an expect value threshold of 0.005 was employed. At the protein level a significance threshold (p) of 0.01 was employed. The described settings resulted in a false discovery rate of 1.6% in average. Additional stringent criteria for protein acceptance were subsequently applied by only accepting proteins identified by two unique peptides in each of the three technical replicates.

A semi‐quantitative analysis of the data was performed using spectral counting. After exclusion of proteins that did not fulfil the criteria described above, the total number of spectral counts (peptide matches) for each identified protein were normalized by the molecular weight of the protein (adjusted matches) and the total value of the adjusted matches for each analysis was used to calculate a percentage abundance of each protein (normalized adjusted matches). Finally, the average abundance (and standard deviation, SD) in each sample was calculated from the three technical replicates. The full LC‐MS/MS results are given in Table S3.

### Genomic libraries

Genomic DNA was partially digested by 0.025–0.25 U/μl ApaLI and NsiI by incubating at 37°C for 15 min. The digested DNA was separated overnight on a 1% SeaPlaque^™^ agarose preparative gel (Lonza Bioscience, BioNordika Denmark A/S, Herlev, Denmark) and high‐molecular weight DNA (8–10 kb) was purified by agarose gel digestion in GELase^™^ enzyme (Epicentre, Madison, WI, USA) followed by standard DNA precipitation. Purified DNA was ligated into the shuttle BAC vector pGNS‐BAC (Kakirde *et al*., 2011) containing a modified multiple cloning site (Vester *et al*., 2014). The vector was transformed into *E. coli* and the libraries were arranged in 96‐well microtiter plates as described (Vester *et al*., 2014). A total of 991 and 1630 clones were picked out for *A. ikkense* and MJ3, respectively, which with an average insert size of 10 kb resulted in a genome coverage of 2.4‐ and 4.3‐fold for the two libraries. Before enzyme screening, libraries were transferred to LB agar plates pH 7.5 containing 12.5 μg/ml chloramphenicol, 2% arabinose, and 0.5 mg/ml AZCL casein and grown overnight at 37°C. Protease activity was then detected by incubation at 20°C. For detection of protease activity at pH 10, plates were grown overnight at 37°C and then overlaid with a thin layer of 0.8% water agar buffered to pH 10 containing 0.5 mg/ml AZCL casein. Vector DNA was extracted by a standard plasmid preparation method, sequenced from both ends and compared to the genome sequence to identify the cloned region.

### Production and characterization of recombinant proteases

Protease‐encoding sequences including the predicted signal peptide were amplified from genomic DNA and cloned into the XhoI and NdeI sites in the expression vector pET21a (*A. ikkense* peg.1776) or pET22b (MJ3 peg.906) with a C‐terminal 6× histidine‐tag. The vectors carrying peg.1776 and peg.906 were transformed into *E. coli* BL21 (DE3) (Life Technologies Europe, Naerum, Denmark) and ArcticExpress (DE3) (Agilent Technologies, Santa Clara, CA, USA) respectively. Clones were initially screened for extracellular protease production on LB agar plates containing AZCL casein. Positive clones were grown in LB medium containing 100 μg/ml ampicillin and expression was induced at an OD of 0.6 by addition of 0.5 mM IPTG after cooling the culture to 20°C. Cultures expressing *A. ikkense* peg.1776 were incubated at 20°C for 20 h and MJ3 peg.906 cultures were incubated at 10°C for 48 h. Cell‐free growth medium containing the expressed protein was harvested by two rounds of centrifugation, sterile‐filtered and concentrated by ultrafiltration. The histidine‐tagged recombinant proteases were affinity purified on a 5 ml HisTrap FF column (GE Healthcare Life Sciences, Brondby, Denmark). The bound proteins were eluted by a linear gradient of 20–500 mM imidazole in 20 mM phosphate buffer pH 7.1, 500 mM NaCl at a flow rate of 2 ml/min. Fractions showing protease activity were collected and stored at 4°C.

Protease activity was assayed on the soluble dye‐linked substrate azocasein (Megazyme) at a concentration of 10 mg/ml according to the manufacturer's protocol. The stability of the recombinant proteases in the assay mixture was confirmed before analysis and assay times were kept short to minimize stability problems, particularly at higher temperatures. All assays were run in a total volume of 100 μl with 5 mM CaCl_2_ and were terminated by addition of three volumes of 5% TCA. Activity was determined by measuring the absorbance of the supernatant at 440 nm after addition of an equal volume 2 M NaOH. The pH optimum was determined in 100 mM three‐component buffer (0.1 M acetic acid, 0.1 M MES, 0.2 M Tris; pH 5–8.5) or glycine buffer (pH 8.5–10.5). Culture supernatant was assayed by incubation at 30°C for 40 min and the recombinant proteases by incubating for 10 min at 40 or 45°C for peg.906 and peg.1776 respectively. Temperature optimum was determined in 100 mM glycine buffer pH 9.5 and reactions were incubated at varying temperatures for 40 min for culture supernatant assays and 10 min for the recombinant proteases.

## Supporting information


**Fig. S1.** The effect of temperature and pH on the growth rate of *Arsukibacterium* sp. MJ3 in R2 broth. The temperature (solid line) and pH measurements (dashed line) were carried out at pH 9 and 15°C respectively.

**Table S1.** Statistics for the genome assemblies of *Arsukibacterium ikkense* (type strain GCM72) and *Arsukibacterium* sp. MJ3 used in this study. Coding sequences and RNAs were predicted by the RAST server (http://rast.nmpdr.org/).

**Table S2.** All proteases predicted from the genome sequences of *Arsukibacterium ikkense* and *Arsukibacterium* sp. MJ3. Proteases were identified by comparison of predicted coding sequences to the Pfam protein domain database (http://pfam.sanger.ac.uk). Signal peptides were predicted by SignalP version 4.1 (http://www.cbs.dtu.dk/services/SignalP/) and results are given as Yes/No, depending on the SignalP score (default cut‐off value of 0.5). Subcellular localization was predicted by PSORTb version 3.0.2 for Gram‐negative bacteria (http://www.psort.org/). The Exoproteome column indicates proteases identified (+) in the exoproteome. See Table S4 for details.

**Table S3.** Complete list of proteins identified and quantified by LC‐MS/MS.

**Table S4.** Sub‐list of all extracellular proteases detected by LC‐MS/MS. Average abundance (±STD) in each sample of all predicted proteases detected in the growth medium of *Arsukibacterium ikkense*. See Table S2 for prediction details. The functional predictions were taken from the RAST server annotations (http://rast.nmpdr.org/).
