# Supplementary material for: Genomic and exoproteomic analyses of cold‐ and alkaline‐adapted bacteria reveal an abundance of secreted subtilisin‐like proteases
Source: Microb Biotechnol. 2016 Feb 1;9(2):245–56. doi: 10.1111/1751-7915.12343 (PMC4767292; doi:10.1111/1751-7915.12343)
Supplement: Supplementary file 1 — Fig. S1. The effect of temperature and pH on the growth rate of Arsukibacterium sp. MJ3 in R2 broth. The temperature (solid line) and pH measurements (dashed line) were carried out at pH 9 and 15°C respectively. [file MBT2-9-245-s001.pdf]

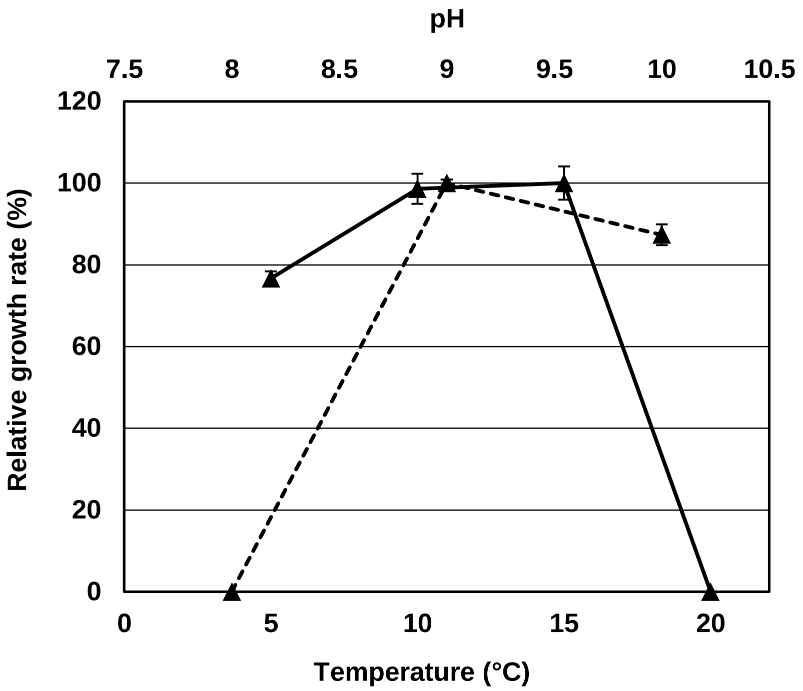

Figure S1. The effect of temperature and pH on the growth rate of *Arsukibacterium* sp. MJ3 in R2 broth. The temperature (solid line) and pH measurements (dashed line) were carried out at pH 9 and 15°C, respectively.
