# Supplementary material for: Genomic and exoproteomic analyses of cold‐ and alkaline‐adapted bacteria reveal an abundance of secreted subtilisin‐like proteases
Source: Microb Biotechnol. 2016 Feb 1;9(2):245–56. doi: 10.1111/1751-7915.12343 (PMC4767292; doi:10.1111/1751-7915.12343)
Supplement: Supplementary file 2 — Table S1. Statistics for the genome assemblies of Arsukibacterium ikkense (type strain GCM72) and Arsukibacterium sp. MJ3 used in this study. Coding sequences and RNAs were predicted by the RAST server (http://rast.nmpdr.org/). [file MBT2-9-245-s002.pdf]

	A. ikkense	*A.* **sp. MJ3**
Contigs	89	196
Min length	231 bp	400 bp
Max length	291,122 bp	329,379 bp
Mean length	46,521 bp	19,114 bp
N50	100,472 bp	146,257 bp
Genome size	4,140,445 bp	3,746,433 bp
Coding sequences	3,818	3,440
RNA	67	63

Table S1. Statistics for the genome assemblies of *Arsukibacterium ikkense* (type strain GCM72) and *Arsukibacterium* sp. MJ3 used in this study. Coding sequences and RNAs were predicted by the RAST server (http://rast.nmpdr.org/).
